# Hypertensive disorder prediction among first-trimester antenatal patients using Gestosis score

**DOI:** 10.6026/973206300210618

**Published:** 2025-04-30

**Authors:** Aashamika P Kundargi, Jagrati K Naagar, Roopali Jain, Nitu Mishra, Priyanka Tiwari

**Affiliations:** 1 Department of Obstetrics and Gynaecology, Government Bundelkhand Medical College, Sagar, Madhya Pradesh, India; 2 Private Practitioner, Sagar, Madhya Pradesh, India; 3 Department of Research Scientist, Multidisciplinary Research Unit, Govt Bundelkhand Medical College, Sagar, Madhya Pradesh, India; 4 Govt Bundelkhand Medical College, Sagar, Madhya Pradesh, India

**Keywords:** Hypertensive disorders of pregnancy gestosis score, hypertensive disorders of pregnancy, pre-eclampsia, risk prediction, maternal health, antenatal screening

## Abstract

The hypertensive disorders of pregnancy Gestosis Score is a cost-effective tool designed to predict hypertensive disorders of pregnancy (HDP), including pre-eclampsia, using maternal history, clinical parameters and laboratory data. Hence, a study of 100 first-trimester antenatal patients found that a Gestosis Score of ≥3 had a sensitivity of 84.64%, specificity of 96.04% and diagnostic accuracy of 93.00% for predicting pre-eclampsia. Pre-eclampsia developed in 17.23% of participants, with a significant correlation between higher Gestosis Scores and increased risk (p < 0.05). Primigravida women (78%) and younger age groups (21-25 years, 49%) were predominant in the study population. The hypertensive disorders of pregnancy Gestosis Score are a reliable and practical screening tool, particularly beneficial in resource-limited settings for early identification of high-risk pregnancies and timely intervention to improve maternal and fetal outcomes.

## Background:

Hypertensive disorders of pregnancy (HDP), including pre-eclampsia, are a significant concern in obstetric care and remain one of the leading causes of maternal and perinatal morbidity and mortality worldwide. According to the World Health Organization, a woman succumbs to complications arising from hypertensive disorders of pregnancy every seven minutes globally [1]. In India, the occurrence of pregnancy-induced hypertension (PIH) is reported to be approximately 10.3%, underscoring the public health burden associated with this condition [2]. Early detection and effective management of hypertensive disorders are, therefore, critical in improving maternal and neonatal outcomes. Pregnancy-induced hypertension is defined as new-onset hypertension (systolic blood pressure ≥140 mmHg and/or diastolic blood pressure ≥90 mmHg) occurring after 20 weeks of gestation in a previously normotensive patient, with or without proteinuria (≥300 mg in 24 hours), resolving within 12 weeks postpartum [3]. It also encompasses cases of new-onset proteinuria in hypertensive patients who were not proteinuric before 20 weeks of gestation. Among these, pre-eclampsia, characterized by hypertension and end-organ dysfunction or proteinuria, represents one of the most severe complications, often leading to adverse maternal and fetal outcomes if not identified and managed early [4]. Various studies have attempted to identify risk factors for pre-eclampsia, such as maternal age, parity, body mass index (BMI), personal or family history of pre-eclampsia and comorbidities like diabetes or polycystic ovarian syndrome (PCOS). However, there is no universally accepted tool that can comprehensively assess and predict the risk of developing hypertensive disorders of pregnancy early in gestation [5, 6].

Recognizing this gap, the hypertensive disorders of pregnancy gestosis score was developed as a novel, evidence-based tool that incorporates both maternal history and emerging risk factors to assess the likelihood of hypertensive disorders of pregnancy development during pregnancy. The hypertensive disorders of pregnancy gestosis score [7], aims to identify high-risk patients during the first trimester. This predictive tool considers a combination of demographic, clinical and biochemical parameters, categorizing patients into mild, moderate, or high-risk groups. By identifying patients at higher risk early in pregnancy, clinicians can implement targeted interventions, including closer monitoring, lifestyle modifications and prophylactic treatments such as low-dose aspirin, to mitigate adverse outcomes [8, 9]. Therefore, it is of interest to describe the hypertensive disorder prediction among first-trimester antenatal patients using Gestosis score.

## Materials and Methods:

The study was done in the Department of Obstetrics and Gynecology at Bundelkhand Medical College, Sagar. It was designed as a prospective observational study, ensuring the collection of comprehensive data over time while minimizing biases. The study methodology adhered to ethical standards and the institutional ethics committee granted approval. The sample size for the study was calculated based on a prevalence of hypertensive disorders in pregnancy of 7.4%, as reported in previous studies, with a 95% confidence interval and a sampling error set at 5%. Using these parameters, the required sample size was determined to be 90 participants. To adjust for 10% loss to follow-up and exclusions based on the study's criteria, the final adjusted sample size was also set at 100 participants. This calculation ensured that the study had adequate statistical power to validate its outcomes.

## Inclusion and Exclusion criteria:

The inclusion criteria for the study focused on antenatal patients in their first trimester of pregnancy. Patients in their second or third trimesters were excluded to maintain homogeneity and focus on early prediction of hypertensive disorders. Additionally, patients with COVID-19, malignancies, liver dysfunction, smoking, or drug abuse were excluded from the study population. These criteria ensured the recruitment of a uniform sample and minimized confounding factors.

## Data collection and variables:

Data collection was comprehensive and involved multiple dimensions of patient history, clinical examinations and laboratory investigations. During the initial visit, participants were interviewed face-to-face and their demographic details, such as name, age, marital status and parity, were recorded. Detailed obstetric history was gathered to identify previous occurrences of hypertensive disorders or pre-eclampsia in prior pregnancies. Medical history was also assessed to determine the presence of conditions like polycystic ovarian syndrome (PCOS), diabetes mellitus and hypertension and thyroid disorders. A detailed surgical history and the use of medications were also documented. Blood pressure readings were taken using a mercury sphygmomanometer with the patient seated upright and the arm positioned at heart level. Mean arterial pressure (MAP) was calculated using the formula: MAP=Diastolic BP+1/3(Systolic BP-Diastolic BP).

A venous blood sample of 5 mL was collected during the antenatal visit (starting at 11 weeks of gestation) to assess complete blood counts, thyroid profiles, blood sugar levels, coagulation parameters and blood grouping. Gestational diabetes mellitus (GDM) was screened by administering a 75 g oral glucose load, irrespective of fasting status, followed by a random blood sugar measurement two hours later. A value of RBS ≥140 mg/dL was used to diagnose GDM. Other conditions, such as systemic lupus erythematous (SLE), were assessed using the 1997 ACR criteria, which require the presence of four out of eleven diagnostic features, including malar rash, renal disease, or neurologic manifestations. Thyroid disorders were identified based on symptoms and laboratory results, with TSH and free T4 levels guiding the diagnosis of hypo- or hyperthyroidism. PCOS was diagnosed using the Rotterdam criteria, requiring the presence of at least two out of three features: hyperandrogenism, ovulatory dysfunction and polycystic ovaries on ultrasonography.

## HDP gestosis score calculation:

The hypertensive disorders of pregnancy gestosis score was calculated manually or through a dedicated mobile application. Risk factors, such as maternal age, BMI, history of cardiovascular diseases and prior hypertensive disorders, were assigned scores ranging from 1 to 3. The scores were then summed up and participants were categorized into three risk groups: mild (score of 1), moderate (score of 2) and high risk (score of 3 or more) for the development of pre-eclampsia. This scoring system incorporated clinical and laboratory parameters to create a comprehensive risk assessment.

## Inclusion of gestosis score components:

The hypertensive disorders of pregnancy gestosis score comprehensively evaluated risk factors, including maternal age, BMI, prior hypertensive disorders, autoimmune diseases and PCOS. These parameters were derived from patient histories, clinical assessments and laboratory investigations. The scoring system was applied uniformly to all participants, ensuring standardized risk stratification.

## Results:

The age distribution of the study participants indicated that the majority of women belonged to the 21-25 years age group, comprising 49% (n=49) of the total sample. Women aged ≤20 years accounted for 40% (n=40), while participants in the 26-30 years age group formed 8% (n=8) and only 3% (n=3) were over 30 years of age. The mean age of the participants was 28.4 ± 6.8 years, reflecting the younger antenatal population attending the outpatient department as seen in Table 1. The majority of participants were Primigravida, accounting for 78% (n=78), while 18% (n=18) were para 1 and 4% (n=4) were para 2 as seen in Table 2. This finding aligns with the established literature, where primi-gravidity is recognized as a significant risk factor for hypertensive disorders of pregnancy.

## Distribution by hypertensive disorders of pregnancy gestosis score:

The hypertensive disorders of pregnancy gestosis score stratified the participants into three risk categories based on the likelihood of developing hypertensive disorders of pregnancy:

[1] Mild Risk (score = 1): 35% (n=35)

[2] Moderate Risk (score = 2): 49% (n=49)

[3] High Risk (score ≥3): 16% (n=16)

The majority of participants (49%) were classified as moderate risk, while 16% were at high risk as seen in Table 3. These classifications underline the tool's ability to categorize patients effectively for risk stratification. The progression of hypertensive disorders was monitored through sequential antenatal visits. At the second visit, 47% (n=47) of participants showed evidence of hypertensive disorders of pregnancy. This increased to 64% (n=64) at the third visit and reached 65% (n=65) at the final visit or time of admission as seen in Table 4. These trends demonstrate the dynamic and progressive nature of hypertensive disorders during pregnancy. The predictive performance of the hypertensive disorders of pregnancy gestosis score in identifying high-risk cases of pre-eclampsia was evaluated using standard metrics. A score of ≥3 showed a sensitivity of 84.64% and a specificity of 96.04%. The positive predictive value (PPV) was 86.80%, while the negative predictive value (NPV) was 85.71%. The overall accuracy of the hypertensive disorders of pregnancy gestosis score in predicting pre-eclampsia was 93.00% as seen in Table 5.

A gestosis score of ≥2 demonstrated higher sensitivity (94%) for detecting hypertensive disorders of pregnancy cases, but the specificity dropped to 49%. Conversely, a score of ≥3 exhibited a high specificity (97.51%), making it more effective at ruling out low-risk cases. These findings emphasize that a higher gestosis score is particularly valuable in accurately identifying patients at significant risk of developing pre-eclampsia. The results demonstrate the hypertensive disorders of pregnancy gestosis score's utility as a screening tool for hypertensive disorders in pregnancy. The score effectively stratifies patients into mild, moderate and high-risk categories, enabling early interventions for those at the greatest risk. The progressive increase in hypertensive disorders of pregnancy cases across antenatal visits emphasizes the need for on-going monitoring and timely management. The high specificity and predictive value of a score of ≥3 further validate its reliability as a diagnostic tool for ruling out low-risk cases.

## Discussion:

The findings of this study reinforce the critical need for effective screening tools to predict pre-eclampsia (PE) and other hypertensive disorders of pregnancy (HDP). Currently, there is no single universally effective screening test for use in the general obstetric population. The International Federation of Gynecology and Obstetrics (FIGO) recommends a first-trimester combined screening test that integrates maternal risk factors with biomarkers such as mean arterial pressure (MAP), serum placental growth factor (PLGF) and uterine artery pulsatility index (UTPI). However, in settings where the measurement of PLGF and UTPI is impractical, FIGO suggests using a combination of maternal risk factors and MAP as the baseline screening approach [10]. Although advanced biomarkers like sFlt1, serum uric acid, TNF-alpha and placental growth factors are highly predictive of PE, their utility is often constrained by high costs, the need for specialized equipment and expertise. This study addresses the limitations of these approaches by evaluating the hypertensive disorders of pregnancy Gestosis Score-a simpler, cost-effective and user-friendly method to stratify risk and predict PE. The findings underscore its potential as a practical tool for widespread application, particularly in resource-limited settings.

## Comparison of age and parity distribution:

In this study, the majority of participants (49%) belonged to the age group of 20-25 years, followed by those aged ≤20 years (40%). These findings are consistent with the demographic profile of antenatal populations in many developing countries. The study also showed a higher prevalence of primigravida patients (65%), aligning with existing evidence that primigravidity is a significant risk factor for the development of hypertensive disorders of pregnancy [11, 12]. Similar demographic trends have been observed in studies from Sweden and China, where the prevalence of pre-eclampsia was reports at 3.98% and 4.02%, respectively [11]. In contrast, studies in developing countries, including India, have shown higher prevalence rates. For instance, Mishra *et al.* reported a prevalence of 15.4% among Indian women [12] and Panda *et al.* identified a prevalence of 7.4% in their study conducted in Shillong and Manipal [13].

## Predictive value of hypertensive disorders of pregnancy gestosis score:

The hypertensive disorders of pregnancy Gestosis Score demonstrated a sensitivity of 84.64%, specificity of 95.03%, positive predictive value (PPV) of 86.80%, negative predictive value (NPV) of 85.71% and diagnostic accuracy of 93.00% for predicting PE. These findings are comparable to a study by Gupta *et al.* which reported a sensitivity of 83.1%, specificity of 97.51%, PPV of 85.51% and diagnostic accuracy of 95.35% [15]. The high specificity of the hypertensive disorders of pregnancy Gestosis Score (≥3) indicates its utility in ruling out low-risk cases, making it particularly valuable for identifying patients who may require more intensive monitoring and early intervention.

## Comparison with existing literature:

Manhar *et al.* in 2023 concluded that the FOGSI Gestosis Scoring System effectively identifies high-risk pregnancies and serves as a practical tool to mitigate the adverse effects of pre-eclampsia through early intervention [16]. Similarly, studies conducted in Ethiopia reported a pre-eclampsia prevalence of 7.9%, emphasizing the importance of early screening to improve maternal and fetal outcomes [14]. The high burden of pre-eclampsia in such settings further emphasizes the importance of predictive tools that allow for timely medical intervention and resource allocation in antenatal care programs. Similarly, the studies conducted by Upadhyay *et al.* [17] and Khanijo *et al.* [18]. The results of this study are in agreement with these findings, highlighting the hypertensive disorders of pregnancy Gestosis Score as a reliable screening method with strong predictive ability. These studies collectively indicate that structured scoring mechanisms contribute to improved risk stratification, reducing the incidence of severe complications associated with hypertensive pregnancy disorders.

## Implications for clinical practice:

The findings of this study demonstrate the practical utility of the hypertensive disorders of pregnancy Gestosis Score in predicting the development of hypertensive disorders of pregnancy. Its simplicity and ease of use make it a feasible option for large-scale implementation, particularly in resource-limited settings where advanced biomarker testing may not be accessible. The score provides a valuable opportunity for obstetricians to identify at-risk patients early, enabling timely intervention and reducing maternal and fetal morbidity and mortality. The changing trends in maternal mortality, with hypertensive disorders overtaking postpartum hemorrhage as a leading cause, underscore the urgent need for such predictive tools. By enabling early detection and appropriate management, the hypertensive disorders of pregnancy Gestosis Score can play a pivotal role in enhancing maternal and neonatal outcomes, particularly in high-burden settings.

## Conclusion:

The hypertensive disorders of pregnancy Gestosis Score is a promising tool for the prediction of pre-eclampsia and hypertensive disorders of pregnancy. Its high sensitivity and specificity, combined with its affordability and simplicity, make it an effective alternative to more complex and costly screening methods. Future research should focus on validating this tool in diverse populations and integrating it into routine antenatal care practices.

## Figures and Tables

**Table 1:** Age distribution of the study population

**Age (years)**	**Frequency**	**Percent (%)**
≤20	40	40
21-25	49	49
26-30	8	8
>30	3	3
Total	100	100

**Table 2:** Parity distribution of the study population

**Parity**	**Frequency**	**Percent (%)**
P0	78	78
P1	18	18
P2	4	4
Total	100	100

**Table 3:** Development of hypertensive disorders in pregnancy (HDP)

**HDP Gestosis Score**	**Interpretation**	**Frequency**	**Percent (%)**
1	Mild Risk	35	35
2	Moderate Risk	49	49
≥3	High Risk	16	16
Total		100	100

**Table 4:** Progression of hypertensive disorders

**Visit**	**Frequency**	**Percent (%)**
2nd ANC visit	47	47
3rd ANC visit	64	64
Final Visit/Admission	65	65

**Table 5:** Predictive ability of hypertensive disorders of pregnancy gestosis score

**Parameter**	**Value**	**95% CI**
Sensitivity	84.64%	71.91% to 95.59%
Specificity	96.04%	85.50% to 95.44%
Positive Predictive Value	86.80%	74.02% to 87.49%
Negative Predictive Value	85.71%	71.82% to 93.39%
Accuracy	93.00%	72.76% to 95.08%
